# Understanding unconventional magnetic order in a candidate axion insulator by resonant elastic x-ray scattering

**DOI:** 10.1038/s41467-023-39138-5

**Published:** 2023-06-09

**Authors:** Jian-Rui Soh, Alessandro Bombardi, Frédéric Mila, Marein C. Rahn, Dharmalingam Prabhakaran, Sonia Francoual, Henrik M. Rønnow, Andrew T. Boothroyd

**Affiliations:** 1 Institute of Physics, École Polytechnique Fédérale de Lausanne (EPFL), Lausanne, Switzerland; 2 Diamond Light Source Ltd., Harwell Science and Innovation Campus, Didcot, Oxfordshire UK; 3 Institute for Solid State and Materials Physics, Technical University of Dresden, Dresden, Germany; 4 Department of Physics, University of Oxford, Clarendon Laboratory, Oxford, UK; 5 Deutsches Elektronen-Synchrotron DESY, Hamburg, Germany

**Keywords:** Topological matter, Magnetic properties and materials

## Abstract

Magnetic topological insulators and semimetals are a class of crystalline solids whose properties are strongly influenced by the coupling between non-trivial electronic topology and magnetic spin configurations. Such materials can host exotic electromagnetic responses. Among these are topological insulators with certain types of antiferromagnetic order which are predicted to realize axion electrodynamics. Here we investigate the highly unusual helimagnetic phases recently reported in EuIn_2_As_2_, which has been identified as a candidate for an axion insulator. Using resonant elastic x-ray scattering we show that the two types of magnetic order observed in EuIn_2_As_2_ are spatially uniform phases with commensurate chiral magnetic structures, ruling out a possible phase-separation scenario, and we propose that entropy associated with low energy spin fluctuations plays a significant role in driving the phase transition between them. Our results establish that the magnetic order in EuIn_2_As_2_ satisfies the symmetry requirements for an axion insulator.

## Abstract (web summary)

EuIn_2_As_2_ is a candidate as an axion insulator, a material that can host axion-like quasi-particles, in direct analogy with the axion proposed in particle physics to resolve the so called “strong CP problem”. Here Soh et al., perform resonant elastic X-Ray scattering on EuIn_2_As_2_ and show that the magnetic order consists of commensurate chiral magnetic structures, satisfying the symmetry requirements for EuIn_2_As_2_ to be an axion insulator.’

## Introduction

One of the key unsolved problems within the framework of the Standard Model of particle physics is how interactions that only involve the strong force preserve the combination of charge conjugation and parity symmetry, even though violations are allowed. The prevailing theory to resolve this strong CP problem invokes a quantum field that permeates all of space^1,2^. Yet, in the intervening 40 years since the first prediction, the excitation of this field, which gives rise to an elementary particle called an axion, has not yet been observed in nature.

Recently, several theoretical studies have predicted that an axion-like field can also occur within certain three-dimensional crystals, where the electronic spectrum is gapped within the bulk and on the surface due to band inversion and magnetic order^3–8^. The study of these axion insulators can not only further our theoretical understanding of axion quasi-particles^9,10^, but also advance technological applications that benefit from a quantized magneto-electric coupling^11–16^.

A prime example is EuIn_2_As_2_, which crystallizes in the centrosymmetric *P*6_3_/*mmc* space group, orders antiferromagnetically below *T*_N_ ≃ 17 K^17–19^, and displays topological signatures in magnetotransport^19,20^. Key to its identification as an axion insulator is the spin configuration of the Eu sublattice, which must be invariant under either an improper rotation or a *C*_*n*_ × *T* symmetry, where *C*_*n*_ is a proper rotation and *T* is the time reversal operation^21^. Density functional theory (DFT) calculations^22–24^ predicted that EuIn_2_As_2_ adopts a collinear *A*-type antiferromagnetic (AFM) order as the ground state spin configuration. This magnetic structure respects *C*_2_ × *T* symmetry and is predicted to stabilize the axion insulator phase.

The magnetic structure of EuIn_2_As_2_ was recently investigated by neutron diffraction,^25^ and instead of A-type AFM order the Eu spins were found to order as a pure helix at *T*_N1_=17.6 K before evolving into a broken helix below *T*_N2_=16.2 K. The proposed temperature evolution for the magnetic structure raises several questions that need to be addressed before EuIn_2_As_2_ can be established as an axion insulator. Firstly, if the periodicity of the broken helix is commensurate with the crystal structure, *C*_2_ × *T* will still be preserved and the axion state protected. However, Ref. ^25^ reports that the size of the broken helix is ~ 3.3 times larger than the structural unit cell along *c*, which breaks the *C*_2_ × *T* symmetry. Secondly, the unpolarized neutron diffraction data alone cannot exclude the possibility of a phase-separated model, with coexisting *A*-type AFM and helical phases in spatially separate regions with different ordering temperatures (*T*_N1_, *T*_N2_). Thirdly, given the importance of the magnetic order of EuIn_2_As_2_ to its axion insulator properties, it is imperative to understand the underlying mechanism driving the symmetry lowering of the magnetic order from the pure to broken helix. Such a mechanism has not yet been proposed.

In this work, we report how the magnetic structure of EuIn_2_As_2_ evolves as a function of temperature and magnetic field by means of resonant elastic x-ray scattering (REXS). We not only confirm the pure and broken helix magnetic phases in EuIn_2_As_2_, but also find that the Eu magnetic structure is commensurate at all temperatures in zero magnetic field and show that the phase-separated model can be excluded. Furthermore, we propose a minimal model which can explain the transition in the magnetic order from pure helix into broken helix. Finally, we determine how the broken helix structure evolves as a function of an in-plane magnetic field.

## Results

### Temperature dependence

**Fig. 1:**
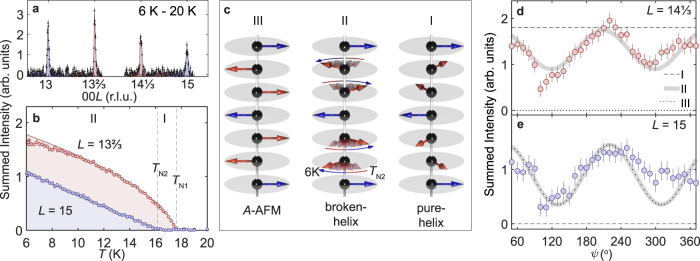
Temperature dependence of the Eu spin configuration. **a** Difference between intensity measurements along 00*L* at *T* = 6 K and 20 K. **b** The integrated intensity of the 00*L* reflections at $L=13\frac{2}{3}$ (red) and 15 (blue). **c** Various magnetic structure models (I–III). Between *T*_N1_ and *T*_N2_, the Eu magnetic sublattice displays a planar helical spin configuration (model I), with a 60^∘^ pitch angle between moments in adjacent basal planes. Below *T*_N2_, the Eu moments undergo a gradual reorientation within the basal plane (broken helix, model II), due to a competition between the pure-helix and the collinear A-type AFM (model III). **d**, **e** Dependence of the intensities of the $L=14\frac{1}{3}$ and 15 reflections measured at *T* = 6 K on the azimuthal angle *ψ*. The dashed, solid and dotted lines are the calculated *ψ* dependence based on models I, II, and III respectively. The error bars correspond to the standard deviation on the integrated intensity of the reflections.

Figure 1a plots the REXS intensity measured at a temperature of *T* = 6 K along the (00*L*) direction in reciprocal space. An identical scan measured in the paramagnetic phase at *T* = 20 K has been subtracted to isolate the magnetic signal. The scans show peaks at integer and non-integer *L*. The non-integer peaks are found at (0, 0, *L*) ± **k**_1_, with *L* an even integer and **k**_1_ = (0, 0, 0.3328(6)). To within experimental error, these peaks are consistent with a commensurate magnetic propagation vector ${{{{{{{{\bf{k}}}}}}}}}_{1}=(0,0,\frac{1}{3})$. The integer peaks belong to a family of 00*L* reflections with odd integer *L* giving a second magnetic propagation vector **k**_2_ = (0, 0, 0). The two propagation vectors were reported previously in Ref. ^25^, but in that study an incommensurate **k**_1_ = (0, 0, 0.303(1)) was found. The distinction between a commensurate and an incommensurate **k**_1_ is important because only a commensurate **k**_1_ can satisfy the *C*_2_ × *T* symmetry needed for the axion insulator phase.

Figure 1b plots the temperature dependence of the integrated intensity of one of each type of magnetic reflection. The peak at $L=13\frac{2}{3}$ follows an order parameter-like temperature dependence below *T*_N1_ = 17.6 K. On further cooling, the integrated intensity of the *L* = 15 peak – which is structurally forbidden by the 6_3_ screw – increases linearly below *T*_N2_ = 16.2 K, without an appreciable anomaly in the integrated intensity of the $L=13\frac{2}{3}$ reflection. Between 6 K and *T*_N2_ we observed that **k**_1_ remains locked to the commensurate value $(0,0,\frac{1}{3})$. In the small interval *T*_N2_ < *T* < *T*_N1_ we observed a slight variation in **k**_1_ with respect to $(0,0,\frac{1}{3})$ of order 1%.

The phase between *T*_N2_ and *T*_N1_, where only **k**_1_-type reflections are observed, can be fully accounted for by the pure spin helix (model I), with a 60^∘^ pitch angle between Eu spins on adjacent basal planes [see Fig. 1c]. According to the model proposed in Ref. ^25^, the **k**_2_-type reflections observed below *T*_N2_ appear because of a gradual in-plane counter-rotation of pairs of Eu moments within the basal plane as shown in model II of Fig. 1c. At *T* = 6 K, the resultant Eu spin configuration is intermediate between the pure helix (model I) and the collinear A-type AFM order (model III).

From the temperature dependence of the $L=13\frac{2}{3}$ and 15 reflections (Fig. 1b) it is not obvious that a distorted period-6 helix would be a superior model to that of coexisting but spatially separated regions of A-type AFM order and pure helical order, respectively. Indeed, these two possibilities cannot be distinguished solely on the basis of unpolarized neutron diffraction data either^25^.

In order to resolve this uncertainty we performed REXS azimuthal scans in which the intensity of x-ray diffraction at the $L=14\frac{1}{3}$ and 15 reflections was measured as a function of *ψ*, the angle of rotation of the sample around the *c* axis. The results are shown in Fig. 1d, e. At $L=14\frac{1}{3}$, the pure spin helix would give a constant intensity as a function of *ψ*, as shown by the dashed line in Fig. 1d, and A-type AFM order would give no intensity at this *L* position. The data, however, displays a sinusoidal *ψ* dependence (with a period of *π*). Therefore, the phase-separated scenario is not consistent with the azimuthal dependence measured at $L=14\frac{1}{3}$ and can be ruled out. The broken helix (model II), on the other hand, does provide a good description of the *ψ* dependence measured at $L=14\frac{1}{3}$ (Fig. 1d), as well as at *L* = 15 (Fig. 1e) where there is no contribution from the helix.

**Fig. 2:**
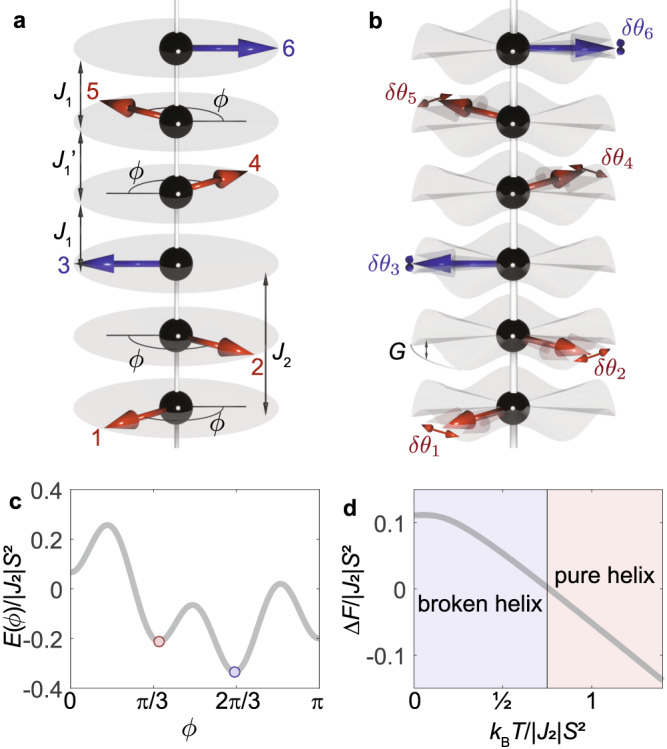
Breaking the pure helix. **a**, **b** Definition of the angle *ϕ*, magnetic exchange energies (${J}_{1},{J}_{1}^{{\prime} }$ and *J*_2_), single-ion anisotropy *G* and in-plane thermal fluctuation (*θ*_*i*_, *i*=1-6). **c** Energy landscape of six europium spins as a function of *ϕ* calculated from eqn (2) with parameters ${J}_{1}=-1.2|{J}_{2}|,{J}_{1}^{{\prime} }=0.5{J}_{1},G{S}^{4}=0.2|{J}_{2}|$, and *J*_2_ < 0. **d** Difference in free energy between the pure helix and the broken helix, where Δ*F* = *F*_PH_ − *F*_BH_.

To shed light on the mechanism which drives the pure helix into a broken helix on cooling below *T*_N2_, we will outline a mean-field model which includes the effect of thermal fluctuations. As the spins are aligned ferromagnetically within the layers it is sufficient to consider the interactions on a chain of spins along the *c* axis. Following Ref. ^25^, we focus on six spins along the chain which we label 1 to 6. We color spins 1, 2, 4 and 5 red, and 3 and 6 blue, as shown in Fig. 2a. The spin configurations of relevance here can be described with a single parameter *ϕ* which defines the in-plane rotation angle of the red spins relative to the blue spins, as shown in Fig. 2a (*ϕ* was called *ϕ*_rb_ in Ref. ^25^). The ideal period-6 helix and the A-type AFM correspond to *ϕ* = *π*/3 and *π*, respectively, and *π*/3 < *ϕ* < *π* describes the broken helix phase observed when *T* < *T*_N2_.

We consider a Heisenberg Hamiltonian with exchange interactions *J*_1_, *J*_2_, ... between first, second, ... nearest neighbors along the chain, and six-fold in-plane spin anisotropy,

1 $${{{{{{{\mathcal{H}}}}}}}}=-\mathop{\sum}\limits_{n}\mathop{\sum}\limits_{k}{J}_{k}{{{{{{{{\bf{S}}}}}}}}}_{n}\cdot {{{{{{{{\bf{S}}}}}}}}}_{n+k}-\mathop{\sum}\limits_{n}G{S}^{6}\cos 6{\theta }_{n},$$

where *n* labels spins on the chain, *θ*_*n*_ is the angle of spin **S**_*n*_ from a local easy direction, and *S* is the Eu spin. As is well known, when *J*_2_ < 0, *G* = 0 and ∣ *J*_1_∣ < 4∣*J*_2_∣ the spin chain is frustrated and the ground state is a pure spin helix with a turn angle between adjacent spins of *τπ*, where $\cos \tau \pi={J}_{1}/4|{J}_{2}|$. In EuIn_2_As_2_ there are two Eu atoms per unit cell along the *c* axis, so the propagation vector is **k** = (0, 0, *τ*). A period-6 helix (*τ* = 1/3) requires *J*_1_ = − 2*J*_2_. Inclusion of *G* > 0 and *J*_3_ < 0 helps to stabilise the period-6 helix.

The broken helix is never a ground state of eqn (1)—it is at best metastable—but one can stabilise the broken helix by dividing the nearest-neighbor exchange into two values, *J*_1_ and ${J}_{1}^{{\prime} }$, corresponding to red–blue and red–red interactions (Fig. 2a). Physically, this could be the result of exchange striction, whose presence would be consistent with the strong spin–lattice coupling observed in optical measurements^26^. With this modification the model can describe all relevant phases for the present system through choice of parameters. Keeping up to second-neighbor exchange in (1), the mean-field energy per spin of the family of period-6 structures described by the parameter *ϕ* is given by

2 $$E(\phi )=	\frac{2}{3}({J}_{2}-{J}_{1}){S}^{2}\cos \phi -\frac{1}{3}({J}_{2}-{J}_{1}^{{\prime} }){S}^{2}\cos 2\phi \\ 	-\frac{1}{3}G{S}^{6}\left[2\cos 6\phi+1\right].$$

As an example, Fig. 2c plots *E*(*ϕ*) for a particular set of parameters that gives a broken helix spin structure with *ϕ* ≃ 2*π*/3 as the ground state.

With this model it is possible to obtain a smooth transition from the broken helix with *ϕ* ≃ 2*π*/3 at low temperature to the pure helix (*ϕ* = *π*/3) on warming up to *T*_N2_, as observed experimentally. However, to accomplish this transition one must vary *J*_1_ over a large range from negative to positive, which is theoretically possible but seems physically unlikely.

We propose instead that the transition is driven (or assisted) by the entropy associated with thermally populated spin fluctuations in a helical state that is metastable at low temperature. Taking the energy curve shown in Fig. 2c as an example, we see that the absolute minimum in the energy is at *ϕ* ≃ 2*π*/3, which will be the ground state at low temperature, but that *E*(*ϕ*) has a local minimum centred on *ϕ* ≃ *π*/3. The curvature at the latter is slightly less than that at the former, and so the density of spin excitations will be higher around *ϕ* ≃ *π*/3 than 2*π*/3. With increasing temperature, the difference in entropy associated with the two minima may drive the system from the broken helix into the pure helix above *T*_N2_.

To demonstrate the plausibility of this mechanism we use the same parameters as before and expand *E*(*ϕ*) to quadratic order around the minima at *ϕ*_0_ ≃ 2*π*/3 and *π*/3. We then calculate the eigenvalues *ω*_*i*_ (*i* = 1 to 6) for a finite chain of six spins, and compute the difference in free energy Δ*F* = *F*_PH_ − *F*_BH_ between the two phases using the expression for the free energy per oscillator of *N* independent quantum harmonic oscillators^27,28^

3 $$F=E({\phi }_{0})-a{k}_{{{{{{{{\rm{B}}}}}}}}}T+\frac{1}{N}\mathop{\sum }\limits_{i=1}^{N}\left\{{k}_{{{{{{{{\rm{B}}}}}}}}}T\ln (1-{{{{{{{{\rm{e}}}}}}}}}^{-\hslash {\omega }_{i}/{k}_{{{{{{{{\rm{B}}}}}}}}}T})+{{\mbox{}}}\frac{1}{2}{{\mbox{}}}\hslash {\omega }_{i}\right\},$$

where *E*(*ϕ*_0_) is the energy at the minimum and *a* is a constant related to the number of degrees of freedom.

The temperature dependence of Δ*F* is plotted in Fig. 2d. We observe a crossover from broken to pure helix at *k*_B_*T* ~ 0.75∣*J*_2_∣*S*^2^. Although the true exchange and anisotropy parameters for EuIn_2_As_2_ are not known, and we have assumed a highly simplified form for the spin excitation spectrum, our model nevertheless does demonstrate how thermal fluctuations could stabilise the pure helix at elevated temperatures even though the broken helix is the energetically stable ground state.

### Magnetic field dependence

**Fig. 3:**
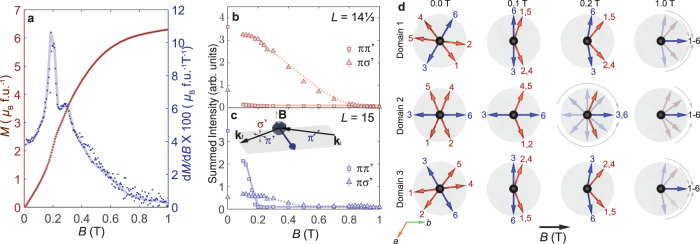
Field dependence of the Eu spin configuration. **a** Magnetization (*M*, red) and the corresponding first derivative (d*M*/d*B*, blue) as a function of field applied along the *b* axis. **b**, **c** Field dependence of the 00*L* reflections at $L=14\frac{1}{3}$ and 15 measured by REXS at *T* = 4 K in the $\pi \to {\pi }^{{\prime} }$ and $\pi \to {\sigma }^{{\prime} }$ scattering channels. (insert) The crystal was oriented with the crystal *b* axis along direction of the applied magnetic field (*B*), which is perpendicular to the horizontal scattering plane. **d** Representation of the field evolution of the Eu spin configurations in the three magnetic domains.

We observe that the magnetization of EuIn_2_As_2_ displays an inflection point at ~ 0.2 T, before gradually increasing towards the fully polarized state (Fig. 3a). Indeed, the first derivative of the magnetization with respect to the applied magnetic field (d*M*/d*B*) displays a sharp peak at the inflection point, indicating an abrupt spin reorientation below 0.2 T.

To understand the origins of these features in the magnetization curves, we consider the field dependence of the *L* = 14$\frac{1}{3}$ and 15 reflections measured by REXS (Fig. 3b, c). Given that the $\pi \to {\pi }^{{\prime} }$ and $\pi \to {\sigma }^{{\prime} }$ scattering channels are probes of the Eu moment components that are parallel and perpendicular to the applied field direction, respectively^29^, we can reconstruct how the Eu spin configuration changes in the external magnetic field.

The dashed and solid lines in Fig. 3b, c were obtained from the structure factor calculation of the *L* = 14$\frac{1}{3}$ and 15 peaks in the $\pi \to {\pi }^{{\prime} }$ and $\pi \to {\sigma }^{{\prime} }$ scattering channels based on the model depicted in Fig. 3d. In zero field, owing to the 120^∘^ rotational symmetry of EuIn_2_As_2_ about the crystal *c* axis, the broken helix has three equivalent magnetic domains with the Eu spins in blue (3 and 6) lying along the three equivalent in-plane easy directions. In a field of 0.1 T applied along the *b* axis, the magnetic domains 1 and 3 adopt a fan-like configuration, whereas the magnetic configuration of domain 2 remains relatively unchanged apart from the red spins 1 and 5 which cant towards the applied field direction.

In intermediate fields (*B* ≃ 0.2 T), where we also observe the inflection point in the magnetization, the intensity of the *L* = 15 peak in the $\pi \to {\pi }^{{\prime} }$ channel goes to 0, whereas the intensity in other scattering channels [$\pi \to {\sigma }^{{\prime} }$ (*L* = 15); $\pi \to {\sigma }^{{\prime} },{\pi }^{{\prime} }$ (*L* = 14$\frac{1}{3}$)] remain relatively unchanged as shown in Fig. 3b, c. This field dependence of the intensity of the 00*L* reflections in both scattering channels can be accounted for by the blue spins in magnetic domain 2 lining up with the direction of the external magnetic field, without significant accompanying changes in the orientation of the red spins, as depicted in the third column of Fig. 3d. Finally, in the high field regime (*B* > 0.2 T), the intensity of both reflections in the $\pi \to {\sigma }^{{\prime} }$ channel decreases gradually to 0 at *B* ~ 1 T, which coincides with the saturation field of the magnetization of EuIn_2_As_2_, Fig. 3a. This is caused by all of the remaining spins canting towards the direction of the applied field, as shown in Fig. 3d, until eventually all the moments are fully polarized.

Our REXS experiments on EuIn_2_As_2_ have established decisively that the magnetic order below *T*_N2_ is a single broken helix structure and not a mixture of two coexisting phases. Magnetization and REXS data as a function of magnetic field also support this picture. Using a mean-field analysis we have found evidence that the unusual broken helix spin structure can be stabilised at low temperatures by two different nearest-neighbour exchange interactions in the *c* direction, and that the transition from the broken helix to the pure helix that occurs on warming above *T*_N2_ is entropically driven.

These results are important because the period-6 helix and broken helix structures both satisfy the symmetry requirements for an axion insulator^25^, which establishes that below its magnetic ordering temperature EuIn_2_As_2_ has the necessary characteristics to realize axion electrodynamics and host the associated non-trivial surface states^23^.

## Methods

### Crystal growth and bulk characterization

The EuIn_2_As_2_ single crystals were grown via a self-flux method similar to that described in Ref. ^17^. The crystals were synthesized from the elements Eu (99.9%), In (99.999%) and As (99.999%), with an excess of indium (In:As molar ratio of 5:1) to serve as the flux. A total of 10 g of the mixture was loaded into an alumina crucible, which was sealed in an evacuated quartz tube. In order to avoid any high arsenic vapour pressure, the sealed quartz tube was slowly heated up to 600 ^∘^C at 25 ^∘^C h^−1^ and held for 5 h. It was further heated up to 950 ^∘^C, then very slowly cooled down to 750 ^∘^C at a rate of 1. 5 ^∘^C h^−1^. The quartz tube was removed from the furnace at 750 ^∘^C and centrifuged to remove the molten indium flux. Shiny crystal in the form of hexagonal platelets with typical dimensions 2 × 2 × 0.2 mm^3^ were obtained. The structure and quality of the crystals was checked on a laboratory 6-circle x-ray diffractometer (Rigaku). The magnetization measurements were performed on a Physical Property Measurement System (Quantum Design) with the vibrating sample magnetometry option, at temperatures down to *T* = 2 K and in magnetic fields of up to *B* = 5 T.

### Resonant elastic X-ray scattering

We performed REXS on single crystalline EuIn_2_As_2_ on beamlines I16 (Diamond^30^) and P09 (PETRA III, DESY^31^) to determine the ground state magnetic order of the Eu spins and its evolution in a magnetic field, respectively. The temperature-dependent REXS experiment on the I16 beamline was performed at the Eu *L*_3_ edge (*E* ≃ 6.973 keV) in the vertical scattering geometry with *π* incident linear polarization achieved with phase plates.

To determine the ground state magnetic configuration, the intensity of the scattered x-rays was measured as function of *ψ*, the angle of rotation of the sample about the scattering vector **Q**. The crystal was aligned with the *c* axis normal to the surface. At each angle *ψ*, the intensity was obtained by summing over the rocking scan of the diffraction peak, with the background subtracted from measurements performed at *T* = 20 *K*. To correct for misalignment of the surface of the crystal, the data was normalized against the fluorescence intensity at each *ψ*. The different magnetic models were tested by calculating the azimuthal dependence from the expressions given in Hill & McMorrow (Ref. ^29^) without any domain-averaging.

The magnetic field-dependent REXS measurements were performed in the second experimental hutch (EH2) of the P09 beamline at the Eu *L*_2_ edge (*E* ≃  7.61 keV) in the horizontal scattering geometry, with incident x-rays of *π* linear polarization. The magnetic field was applied normal to the scattering plane [see insert in Fig. 3c]. The ${\pi }^{{\prime} }$ or ${\sigma }^{{\prime} }$ polarization component of the scattered x-rays was selected using a Cu(220) analyzer crystal. The same EuIn_2_As_2_ crystal was used in both REXS experiments.

## Data Availability

The data presented in this study have been deposited in a Zenodo repository at 10.5281/zenodo.7949497.
